# Borderline Personality Disorder And Childhood Trauma: Witch Relationship?

**DOI:** 10.1192/j.eurpsy.2022.949

**Published:** 2022-09-01

**Authors:** M. Turki, N. Gargouri, M. Abdellatif, S. Ellouze, O. Abidi, N. Halouani, J. Aloulou

**Affiliations:** Hedi Chaker University Hospital, Psychiatry “b” Department, Sfax, Tunisia

**Keywords:** Childhood Trauma, borderline personality disorder

## Abstract

**Introduction:**

Borderline Personality Disorder (BPD) is a pervasive pattern of impulsiveness, emotional dysregulation, and difficult interpersonal relationships. Several studies showed that its onset depends on the combination of biological and psychosocial factors, particularly between biological vulnerabilities and traumatic experiences during childhood.

**Objectives:**

We aimed to explore the mediators of the effects of childhood trauma in BPD vulnerability.

**Methods:**

We conducted a literature review using “PubMed” database and keywords “borderline personality disorder”, “childhood trauma”, “hypothalamic-pituitary-adrenal axis”, “stress”, adverse childhood experiences”.

**Results:**

Several studies showed that a diagnosis of BPD is associated with child abuse and neglect more than any other personality disorders, with a range between 30 and 90% in BPD patients. All types of abuse and neglect happen to be significantly associated with BPD features. Besides, the exposure to multiple types of maltreatment through multiple development periods increased the severity of BPD. Several studies highlighted the role of alterations in Hypothalamic-Pituitary-Adrenal axis, in neurotransmission, in the endogenous opioid system and in neuroplasticity in the childhood trauma-associated vulnerability to develop BPD. Besides, morphological changes in several BPD brain areas and in particular in those involved in stress response have also been incriminated.

**Conclusions:**

Our findings regarding the role of childhood trauma in the development of BPD would help identify and develop early intervention services for a vulnerable population. The critical role of psychotherapy in treating individuals with early life stress may partially explain why the prevailing empirically validated treatments for BPD are psychotherapeutic.

**Disclosure:**

No significant relationships.

